# Analyzing Predominant Bacterial Species and Potential Short-Chain Fatty Acid-Associated Metabolic Routes in Human Gut Microbiome Using Integrative Metagenomics

**DOI:** 10.3390/biology12010021

**Published:** 2022-12-22

**Authors:** Amornthep Kingkaw, Nachon Raethong, Preecha Patumcharoenpol, Narissara Suratannon, Massalin Nakphaichit, Suttipun Keawsompong, Sittiruk Roytrakul, Wanwipa Vongsangnak

**Affiliations:** 1 Interdisciplinary Graduate Program in Bioscience, Faculty of Science, Kasetsart University, Bangkok 10900, Thailand; 2 Institute of Nutrition, Mahidol University, Nakhon Pathom 73170, Thailand; 3 Center of Excellence for Allergy and Clinical Immunology, Division of Allergy, Immunology and Rheumatology, Department of Pediatrics, Faculty of Medicine, Chulalongkorn University, King Chulalongkorn Memorial Hospital, The Thai Red Cross Society, Bangkok 10330, Thailand; 4 Department of Biotechnology, Faculty of Agro-Industry, Kasetsart University, Bangkok 10900, Thailand; 5 Functional Ingredients and Food Innovation Research Group, National Center for Genetic Engineering and Biotechnology, National Science and Technology Development Agency, 144 Thailand Science Park, Phaholyothin Road, Pathum Thani 12120, Thailand; 6 Department of Zoology, Faculty of Sciences, Kasetsart University, Bangkok 10900, Thailand; 7 Omics Center for Agriculture, Bioresources, Food, and Health, Kasetsart University (OmiKU), Bangkok 10900, Thailand

**Keywords:** copra meal hydrolysate, human gut microbiome, metagenomics, metabolism, short-chain fatty acids

## Abstract

**Simple Summary:**

Human gut microbiome plays an important role for health. This study was thus aimed to analyze the predominant species and metabolic routes involved in short-chain fatty acids (SCFAs) production in the human gut microbiome after treatment with copra meal hydrolysate (CMH). Using integrative metagenomics, key predominant bacterial species and metabolic routes involved in cooperative microbiome networks in relation to SCFAs biosynthesis were identified. This suggests that CMH becomes a potential prebiotic diet for modulating and maintaining the gut microbiome implicated in human health.

**Abstract:**

Gut microbiome plays an essential role in host health, and there is interest in utilizing diet to modulate the composition and function of microbial communities. Copra meal hydrolysate (CMH) is commonly used as a natural additive to enhance health. However, the gut microbiome is largely unknown at species level and is associated with metabolic routes involving short-chain fatty acids (SCFAs). In this study, we aimed to analyze, using integrative metagenomics, the predominant species and metabolic routes involved in SCFAs production in the human gut microbiome after treatment with CMH. The effect of CMH treatment on the Thai gut microbiome was demonstrated using 16S rRNA genes with whole-metagenome shotgun (WMGS) sequencing technology. Accordingly, these results revealed that CMH has potentially beneficial effects on the gut microbiome. Twelve predominant bacterial species, as well as their potential metabolic routes, were involved in cooperative microbiome networks under sugar utilization (e.g., glucose, mannose, or xylose) and energy supply (e.g., NADH and ATP) in relation to SCFAs biosynthesis. These findings suggest that CMH may be used as a potential prebiotic diet for modulating and maintaining the gut microbiome. To our knowledge, this is the first study to reveal the predominant bacterial species and metabolic routes in the Thai gut microbiome after treatment with potential prebiotics.

## 1. Introduction

The human gut microbiome contains highly diverse microbial communities that play essential roles in human health. The gut microbiome is influenced by several factors, such as host genetics, diet, lifestyle, medication, and environment [1,2]. In addition, consumption of specific nutritional substances, such as prebiotics, can modulate gut microbial composition [3,4]. Prebiotics include diverse types of oligosaccharides that are subjected to microbial fermentation in the gastrointestinal (GI) tract, and they affect microbial communities and their functions, offering energy and maintaining gut homeostasis [5,6].

Manno-oligosaccharides (MOS) consist of a linear chain of mannose and have gained great interest as a prebiotic. MOS can be derived from mannan-rich plants, such as copra meal, a by-product of coconut milk and coconut oil processing. In Thailand, 25 million metric tons of copra meal is produced annually [7]. By enzymatic hydrolysis using β-mannanase, a copra meal hydrolysate (CMH) was obtained as a source for further MOS production. CMH is stable under human gastrointestinal tract conditions. CMH is relatively stable in the small intestine and is easily fermented by *lactobacilli* and *bifidobacteria* [8]. Very recently, the impact of CMH on gastrointestinal symptoms and gut microbiome was demonstrated to have a positive relationship with health using 16S rRNA gene sequencing [9]. In the context of taxonomy and metabolic function-associated pathways, the human gut microbiome is largely unknown at the species level, as are the associated metabolic routes involved in short-chain fatty acids (SCFAs).

Therefore, this study aimed to analyze the predominant species and metabolic routes involved in SCFAs production in the human gut microbiome after treatment with CMH using integrative metagenomics. Different treatments, such as baseline, placebo, or CMH, on the Thai gut microbiome were demonstrated using 16S rRNA genes and whole-metagenome shotgun (WMGS) sequencing technology. DNA extraction from fecal samples was initially performed, and sequencing data were then analyzed using bioinformatics and systems biology tools and databases for taxonomic profiles and metabolic functions of the gut microbiome. Integrative analysis with 16S rRNA genes and WMGS data revealed a number of predominant bacterial species, as well as a list of potential metabolic functions and associated routes involved in SCFAs production after treatment with CMH. This is the first study to reveal that CMH is a potential prebiotic capable of modulating and maintaining the gut microbiome implicated in human health.

## 2. Materials and Methods

### 2.1. Participants and Fecal Sample Collection

For participants, thirty-seven Thai adults—from Bangkok and its closer city, Thailand—aged between 18–45 years under a BMI of 18.5–24.0 kg/m^2^ were registered with double-blinded, placebo-controlled trials in this cohort. Concerning recruitment, they were informed at King Chulalongkorn Memorial Hospital, Bangkok, Thailand, under stringent inclusion and exclusion criteria i.e., dietary intake, age, and status of health. Notably, the participants had no intestinal diseases or diarrheal symptoms in the months prior to sampling and none of the patients had a family history of colorectal cancer. In addition, the participants had to not receive antibiotics within at least three months as well as probiotics, prebiotics, and synbiotics at least one month before sampling. Participants with allergies to coconuts or food intolerance were excluded. The clinical and demographic characteristics of the study participants in the cohort were described according to Sathitkowitchai et al. (2021) [9] (see Supplementary File S1).

For fecal sample collection, participants were assigned to four different groups in a total of 74 samples: baseline CMH (bCMH for 17 samples), baseline placebo (bPB for 20 samples), treatment with CMH (tCMH for 17 samples), and treatment with placebo (tPB for 20 samples). Remarkably, the baseline means that they were not treated with either CMH or PB. In addition, treatment meant that they were subjected to CMH or PB for 21 days. Fecal samples (20 g) were immediately collected at the time of defecation and placed into a collection tube in a cooler bag. The fecal samples were stored at –80 °C for further analysis.

This study was approved by the Thai Clinical Trials Registry (trial identification number TCTR20190426003) and the Ethics Committee of King Chulalongkorn Memorial Hospital, Bangkok, Thailand (IRB No. 388/61). All methods were performed in accordance with relevant guidelines and regulations. Written informed consent was obtained from all participants.

### 2.2. DNA Extraction and Metagenome Sequencing

All 74 samples were subjected to DNA extraction using a modified method described by Nakphaichit et al. (2014) [10]. Subsequently, 16S rRNA gene sequencing was performed according to the method described by Sathitkowitchai et al. (2021) [9]. Of the 74 samples, 20 were prepared for WMGS sequencing, annotation, and analysis. Initially, fecal samples were centrifuged for 2 min at 13,000 ×g. The pellet was washed twice, centrifuged in phosphate-buffered saline solution (PBS, 1 mL) at 13,000 ×g for 5 min, and then suspended in 900 µL PBS. The supernatant was then discarded. Total DNA purification with quality control assessments and metagenome sequencing was performed according to the method described by Raethong et al. (2021) [11]. Clean reads, which were not mapped to the human genome, were obtained as the metagenomic dataset of the gut microbiome.

### 2.3. Microbial Taxonomic Analysis and Functional Annotation of 16S rRNA Gene Sequencing Data and WMGS Datasets

Initially, 16S rRNA gene sequence datasets (i.e., 17 bCMH samples, 20 bPB samples, 17 tCMH samples, and 20 tPB samples) were analyzed for microbial taxonomy and function using QIIME 2, PICRUSt2 pipeline package (v2.1.0_b) [12], and MetGEMs toolbox (v1.0) [13]. Exploring the MetGEMs toolbox, Core Function was used to investigate the metabolic functions, the KO IDs in each sample were rank-transformed, and the geometric means of KO IDs of each group were then computed.

To analyze WMGS datasets, 20 samples out of 74 samples were randomly selected (5 bCMH samples, 5 bPB samples, 5 tCMH samples, and 5 tPB samples). Alpha- and beta-diversity analyses were initially performed using a vegan package [14] in the R program (version 2.5-6). For alpha-diversity, the observed species and Shannon diversity indices were used to calculate species richness and abundance. Statistical differences in diversity indices between possible pairwise comparison groups were identified using the Wilcoxon rank-sum test. Beta-diversity was computed using Bray–Curtis distances with the metaMDS function in the vegan R package [14]. Beta-diversity analysis was performed to investigate differences between microbial communities across possible pairwise comparison groups. Differences in beta-diversity were visualized through non-metric multidimensional scaling (NMDS) ordination using the ggplot2 R package [15]. Beta-diversity was tested for inference by permutational multivariate analysis of variance (PERMANOVA), as implemented in the ADONIS function from the vegan R package [14], using permutations equal to 999. To test the difference in microbial composition between two or more groups, analysis of similarities (ANOSIM) was also evaluated based on the Bray–Curtis dissimilarity using the vegan R package [14].

For microbial taxonomic analysis, MetaPhlAn (v3.0) [16] was used to detect and quantify individual species with a library of clade-specific markers (mpa_v30_CHOCOPhlAn_201901) as database and to further generate overall metagenome profiles, for example, phyla, families, genera, species, and strain levels. The relative abundance of the microbial taxonomic level was plotted using the ggplot2 package [15] in the R program (v.3.5.3). The Wilcoxon rank-sum test was used to identify statistically significant differences (*p* < 0.1) between possible pairwise comparison groups.

The relative abundance of gene families and pathways was determined using HUMAnN 3. The gene profile was summed into KO IDs and normalized using the built-in script in HUMAnN 3 [17]. To further determine the metabolic function of the gut microbiome, the obtained KO IDs from the 16S rRNA gene sequence and WMGS dataset were searched against the KEGG database using KEGG mapper in KEGG mapping tools [18,19,20]. Once KEGG Orthology (KO) IDs were identified, they were classified into six functional categories: metabolism, genetic information processing, cellular processes, environmental information processing, organism systems, and human diseases. Further, the functional and pathway enrichment analysis was explored under a distinct up-directional *p*-value < 0.05 for the potential group, for example, pairwise comparison between tCMH and tPB.

### 2.4. Identification of Predominant Bacterial Species, Potential Metabolic Functions and Associated Routes Involved in Treatment with CMH Using Integrative Analysis

To identify the predominant bacterial species, potential metabolic functions, and associated routes when treated with CMH, taxonomic profiles, and metabolic functions obtained from 16S rRNA gene sequences and WMGS datasets were integrated. After analyzing taxonomy and metabolic functional as well as pathway enrichments, the predominant bacterial species and enriched KO IDs under positive median mode and *p*-value < 0.05 were then considered. Based on the KEGG database, the targets of enriched KO IDs across predominant species were mapped to metabolic pathways, for example, SCFA biosynthesis, using mapper in KEGG mapping tools. Furthermore, literature mining and manual curation were performed to uncover a number of predominant bacterial species, as well as a list of potential metabolic functions and associated routes.

## 3. Results and Discussion

### 3.1. Assessment of 16S rRNA Gene Datasets on Microbial Composition and Metabolic Function

**Figure 1:**
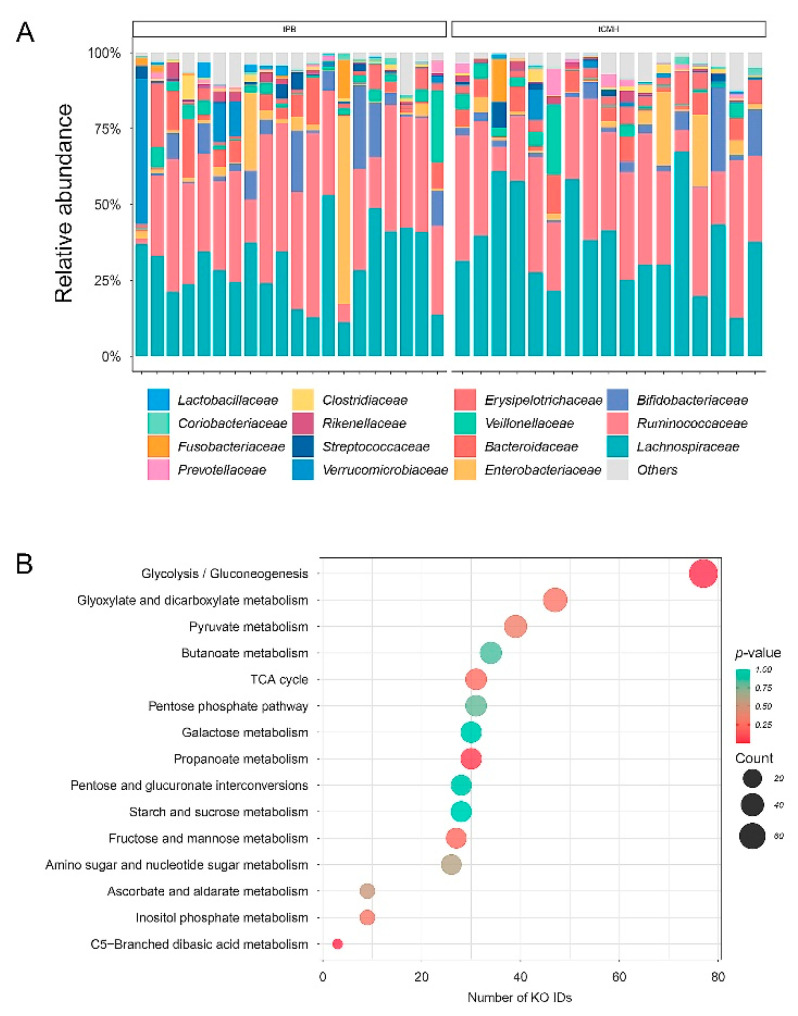
Microbial composition and metabolic function after treatment with CMH, using 16S rRNA gene sequences datasets. (**A**) Microbial composition changes at family level. (**B**) Top 15 associated pathways in carbohydrate metabolism. Note: tPB and tCMH represent treatment with placebo and CMH, respectively.

After treatment of the Thai gut microbiome with CMH (tCMH), the microbial composition in the human gut was assessed using 16S rRNA gene datasets. Four phyla (Firmicutes, Bacteroidetes, Actinobacteria, and Proteobacteria) were most commonly identified. Firmicutes showed the highest relative abundance in the bacterial community, accounting for 80.6%. At the family level, tCMH was found in the top five bacterial families (*Lachnospiraceae*, *Bacteroidaceae*, *Erysipelotrichaceae*, *Prevotellaceae*, and *Rikenellaceae*) under the positive median mode (Figure 1A). *Ruminococcaceae* was reduced in the tCMH group. These results are consistent with those reported by Sathitkowitchai et al., 2021 [9]. Across the other pairwise groups, for example, at the phyla level, the changes in the gut microbiome were similar (see Supplementary File S1).

Metabolic function prediction of the gut microbiome focused on metabolism (Figure 1B). The top three associated pathways involved in carbohydrate metabolism were the major enrichments. Considering the positive median mode under statistical significance (*p* ≤ 0.05), glycolysis/gluconeogenesis, propanoate metabolism, C5-Branched dibasic acid metabolism were targeted in relation to the central carbon metabolism and energy supply. There were target of enzymes e.g., phosphoenolpyruvate carboxykinase (ATP) (EC:4.1.1.49), 3-isopropylmalate dehydrogenase (EC:1.1.1.85), pyruvate, orthophosphate dikinase (EC:2.7.9.1), aldose 1-epimerase (EC:5.1.3.3), fructose-1,6-bisphosphatase II (EC:3.1.3.11), fructose-1,6-bisphosphatase I (EC:3.1.3.11), fructose-1,6-bisphosphatase III (EC:3.1.3.11), fructose-1,6-bisphosphatase I/sedoheptulose-1,7-bisphosphatase (EC:3.1.3.11/3.1.3.37), fructose-1,6-bisphosphatase II/sedoheptulose-1,7-bisphosphatase (EC:3.1.3.11 3.1.3.37), fructose 1,6-bisphosphate aldolase/phosphatase (EC:4.1.2.13/3.1.3.11), pyruvate kinase (EC:2.7.1.40) pyruvate kinase isozymes R/L (EC:2.7.1.40), methylglyoxal synthase (EC:4.2.3.3) (see Supplementary File S1).

### 3.2. Assessment of Gemicrobial Diversity and Composition from WMGS Datasets

Raw WMGS datasets were assessed for 1141.74 Megabases (Mb) of all samples (Table 1). After discarding low-quality reads, adapter and human genome contaminants, a total of 1132.41 Mb of clean reads were retrieved with an average effective rate of 99.18% (see Supplementary File S2) and used for further analysis.

**Figure 2:**
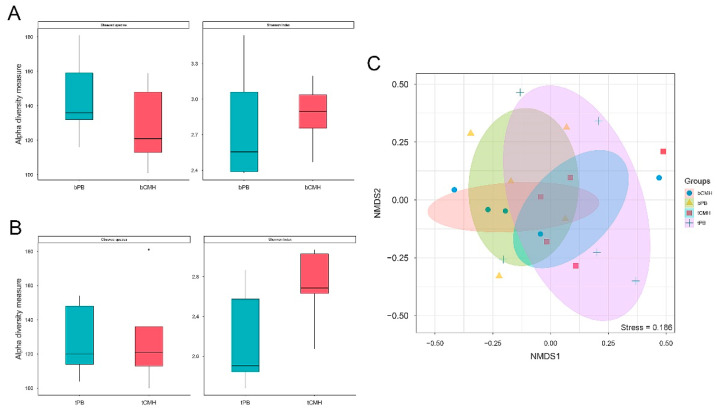
Comparison of microbial diversity across all possible groups. (**A**,**B**) Boxplots of the alpha-diversity in contexts of observed species and Shannon index of each pairwise group, respectively; (**C**) Beta-diversity plot of non-metric multidimensional scaling (NMDS) ordination in context of Bray–Curtis dissimilarity among each group. Taxonomic diversity was assessed at species level. In addition, bPB, bCMH, tPB, and tCMH represent baseline placebo, baseline CMH, treatment with placebo, and treatment with CMH, respectively.

Alpha- and beta-diversity analyses were performed to determine the microbial diversity across all possible groups. The observed species and Shannon diversity indices showed that each group was comparable, as shown in Figure 2A,B. Based on the Wilcoxon rank-sum test, there were no statistically significant differences in alpha-diversity among all groups. Regarding beta diversity, an NMDS plot of the Bray–Curtis dissimilarity index is shown in Figure 2C. The results also showed that beta-diversity was not significantly different across all groups (ANOSIM analysis: R = –0.236, *p* > 0.05; ADONIS analysis: R^2^ = 0.127; *p*-value > 0.05). This indicates that the diversity between the baseline and treatment groups was comparable in the context of taxonomic richness and abundance.

**Figure 3:**
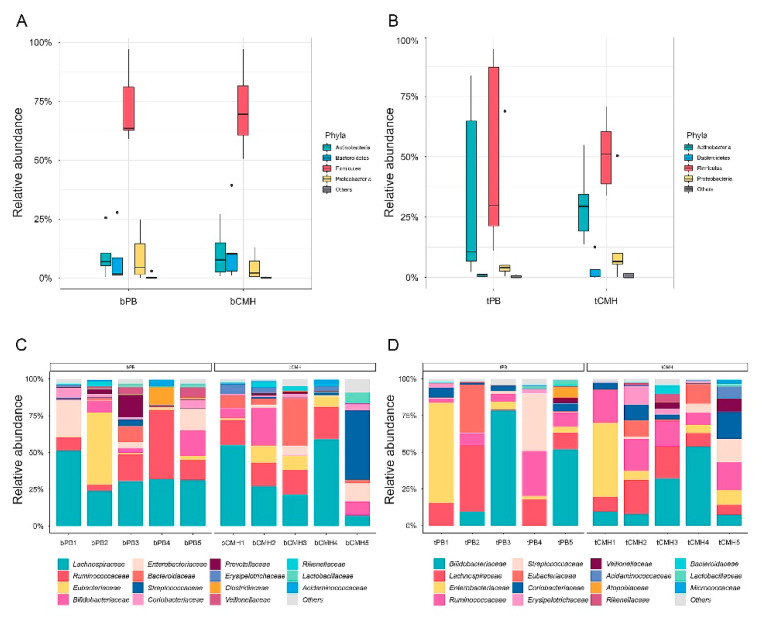
Taxonomic profiles of gut microbiome using WMGS datasets. (**A**,**B**) Boxplot of relative abundance of microbial phyla across baselines and treatments, respectively. (**C**,**D**) Stacked column graph showing the relative abundance of taxonomic families across baselines and treatments, respectively. In addition, bPB, bCMH, tPB, and tCMH represent baseline placebo, baseline CMH, treatment with placebo, and treatment with CMH, respectively.

To initially assess the taxonomic profiles of the gut microbiome, the WMGS datasets were analyzed for all taxa. As presented in Figure 3A,B, we found that four phyla (Firmicutes, Actinobacteria, Proteobacteria, and Bacteroidetes) were comparable across the baselines (bPB and bCMH) and treatments (tPB and tCMH). Among these four phyla, Firmicutes showed the highest relative abundance in the bacterial community for all groups, accounting for an average of 60.99%. The family *Lachnospiraceae*, belonging to the genus *Roseburia* in the phylum Firmicutes, was dominant at a high abundance (23.73%). Considering all groups under the top 15 bacterial families (see Supplementary File S2), five dominant families (Figure 3C,D) were found: *Bifidobacteriaceae*, *Enterobacteriaceae*, *Eubacteriaceae, Lachnospiraceae*, and *Ruminococcaceae*. These results are consistent with those of La-ongkham et al. (2020) [21] in the context of the core taxonomy of the Thai gut microbiome.

**Figure 4:**
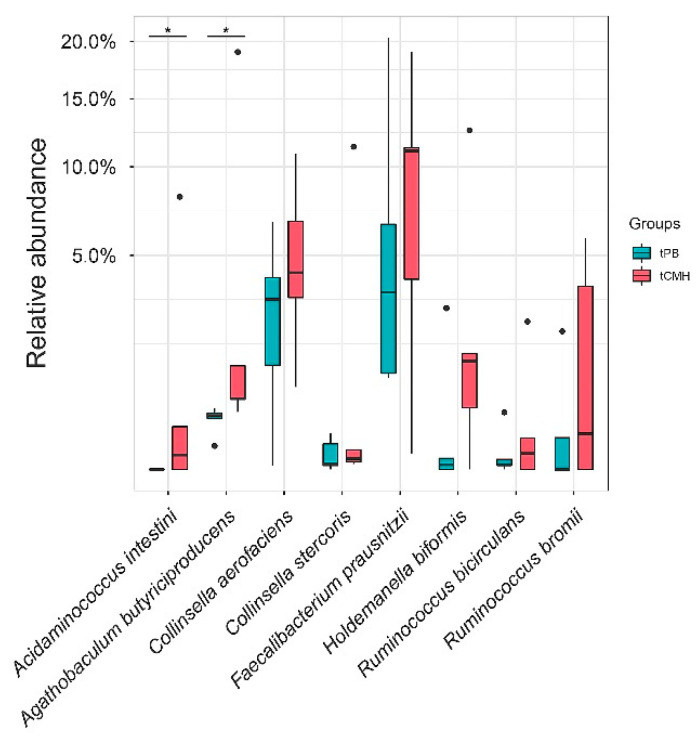
Comparison of relative abundance between tPB and tCMH on eight potential species, using WMGS datasets. For those marked with an asterisk (*), significant difference between tCMH and tPB groups is shown by different letters (*p*-value < 0.1).

Focusing on tCMH, five alternative dominant families—*Ruminococcaceae*, *Coriobacteriaceae*, *Erysipelotrichaceae*, *Veillonellaceae*, and *Acidaminococcaceae*—were observed. Of these families, eight potential species were identified (Supplementary File S2): *Acidaminococcus intestini*, *Agathobaculum butyriciproducens*, *Collinsella aerofaciens*, *Collinsella stercoris*, *Faecalibacterium prausnitzii*, *Holdemanella biformis*, *Ruminococcus bicirculans*, and *Ruminococcus bromii* (Figure 4). Notably, two species (*p*-value < 0.1), *A. intestini* and *A. butyriciproducens*, were significantly involved in metabolic functions.

### 3.3. Assignment of Metabolic Function Underlying KO IDs from WMGS Datasets

**Figure 5:**
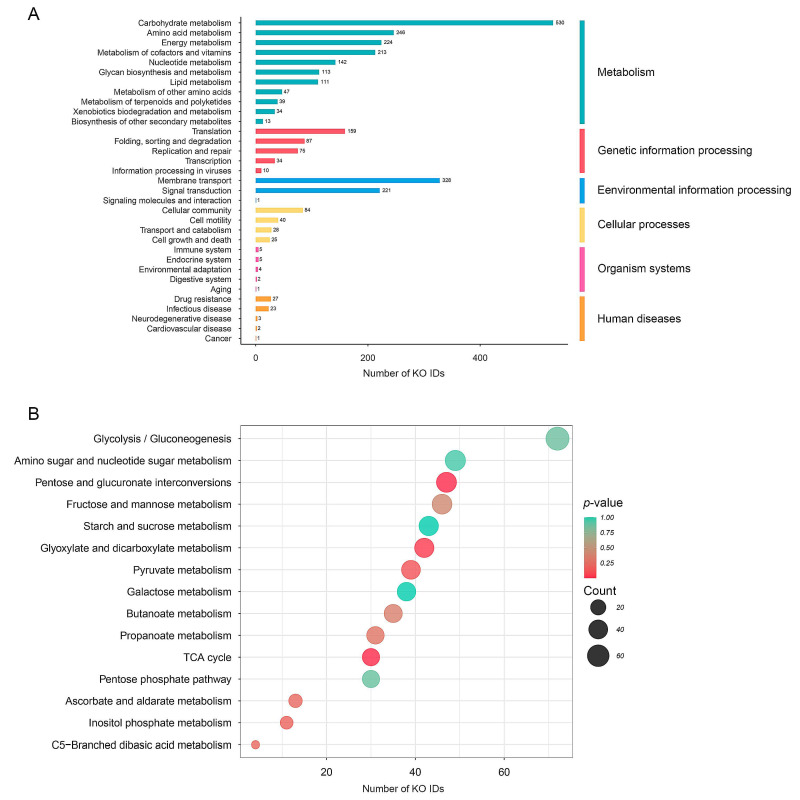
Functional categories underlying KO IDs from WMGS datasets of gut microbiome (**A**) Main functional categories using KEGG (**B**) Sub-metabolic categories in carbohydrate metabolism.

Concerning on all bacterial families achieved from taxonomic profiles (see Supplementary File S2), metabolic functional analysis was performed using KEGG. A total of 5421 KO identifiers (KO IDs) were identified. Across six functional categories, metabolism was shown to have the highest number of KO IDs (1,712 KO IDs), followed by genetic information processing (365 KO IDs), environmental information processing (550 KO IDs), cellular processes (177 KO IDs), organism systems (17 KO IDs), and human diseases (56 KO IDs), as shown in Figure 5A and Supplementary File S2. As observed, 530 out of 1,712 KO IDs were mainly found in carbohydrate metabolism. Genes involved in glycolysis/gluconeogenesis (72 KO IDs), amino sugar and nucleotide sugar metabolism (49 KO IDs), pentose and glucuronate interconversions (47 KO IDs), fructose and mannose metabolism (46 KO IDs), starch and sucrose metabolism (43 KO IDs), glyoxylate and dicarboxylate metabolism (42 KO IDs), pyruvate metabolism (39 KO IDs), galactose metabolism (38 KO IDs), butanoate metabolism (35 KO IDs), propanoate metabolism (31 KO IDs), TCA cycle (30 KO IDs), pentose-phosphate pathway (30 KO IDs), ascorbate and aldarate metabolism (13 KO IDs), inositol phosphate metabolism (11 KO IDs), and C5-branched dibasic acid metabolism (4 KO IDs) (Figure 5B and Supplementary File S2).

**Figure 6:**
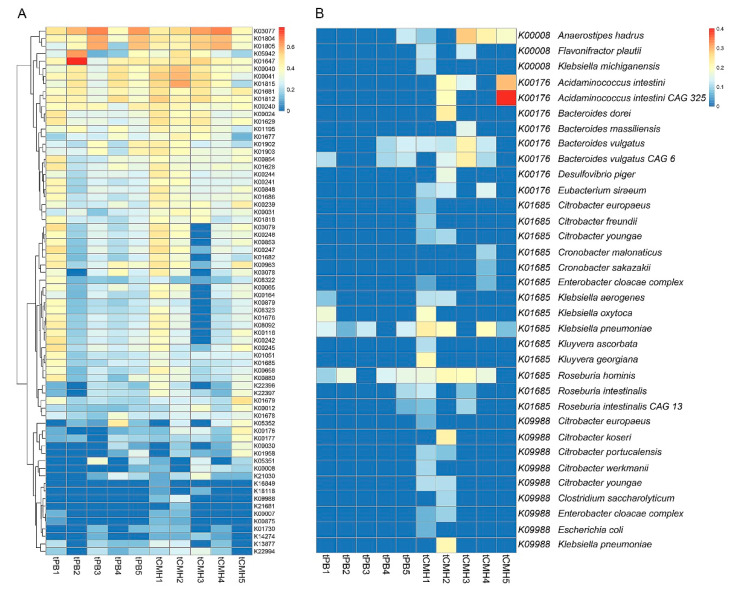
Heatmaps represent the enriched metabolic pathways underlying KO IDs (**A**) KO IDs involved in TCA cycle and pentose and glucuronate interconversions (**B**) Bacterial species underlying significant KO IDs. Note: Color maps display fourth root of RPKM value.

Metabolic function and pathway enrichment analyses were performed for carbohydrate metabolism. Interestingly, we found that the TCA cycle and pentose and glucuronate interconversions were significantly enriched (*p* < 0.05) (Figure 5B and Figure 6A; see Supplementary File S2).

Upon tCMH, four KO IDs were identified: altronate hydrolase (K01685, EC:4.2.1.7), 2-oxoglutarate ferredoxin oxidoreductase subunit delta (K00176, EC:1.2.7.3), l-iditol 2-dehydrogenase (K00008, EC:1.1.1.14), and d-lyxose ketol-isomerase (K09988, EC:5.3.1.15) (Figure 6A) (see Supplementary File S2). These enzymes are essential for 2-oxoglutarate and succinate formation, leading to the biosynthesis of SCFAs. Succinate is a key precursor and plays an important role in either propionate or acetyl-CoA formation, which can then be converted to acetate or butyrate formation [22,23,24].

After SCFA-producing species mapping with the target of four KO IDs, 11 species were identified: Acidaminococcus intestini, Anaerostipes hadrus, Bacteroides dorei, Bacteroides massiliensis, Bacteroides vulgatus, Clostridium saccharolyticum, Desulfovibrio piger, Escherichia coli, Eubacterium siraeum, Roseburia hominis, and Roseburia intestinalis (Figure 6B and Supplementary File S2). These results are consistent with those of Nogal et al. (2021), who showed that SCFAs are produced by enteric and acetogenic commensal microbes, such as Bacteroides spp. and Clostridium spp. [22]. Exhibiting functions involved in succinate formation towards SCFA biosynthesis, for example, propionate, butyrate, and acetate, four out of 11 species (i.e., A. hadrus, R. hominis, A. intestine, and E. siraeum) were interested (Figure 6B) [25,26].

Altogether, our results agree well with earlier reports on propionate/butyrate-producing microbes, such as *A. intestini*, *E. coli, Bacteroides* spp., *Anaerostipes, Eubacterium* and *Roseburia* [27].

### 3.4. Identifying Predominant Bacterial Species and Potential Metabolic Routes Using Integrative Metagenomics

**Figure 7:**
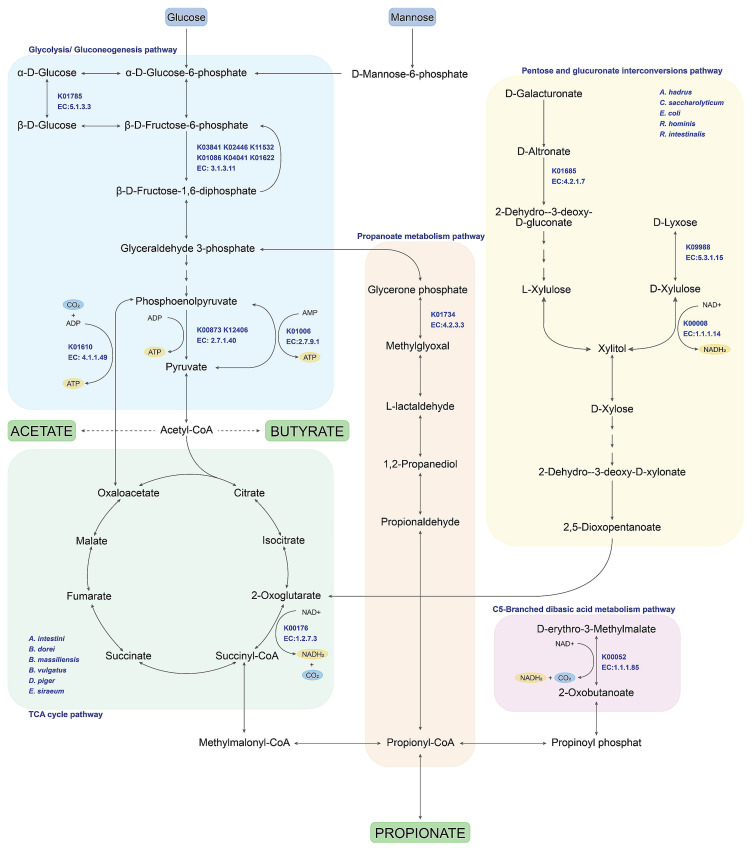
Potential species and related metabolic routes involved in SCFAs biosynthetic pathways. Note: KEGG was used as a pathway database.

To identify the predominant bacterial species, the results obtained from the 16S rRNA gene sequence datasets (Figure 1B) and the WMGS datasets (Figure 6B) were integrated. Post-tCMH, we found 12 predominant species: *A. intestini* and *A. butyriciproducens, A. hadrus*, *B. dorei*, *B. massiliensis*, *B. vulgatus*, *C. saccharolyticum*, *D. piger*, *E. coli*, *E. siraeum*, *R. hominis*, and *R. intestinalis*. According to literature supports for all identified species [28], very interestingly, they were SCFA-producing species. The results of mapping the enriched KO IDs identified from these SCFA-producing species onto SCFA biosynthesis are shown in Figure 7. Indeed, it revealed five metabolic pathways—glycolysis/gluconeogenesis, TCA cycle, pentose and glucuronate interconversions, C5-branched dibasic acid metabolism, and propanoate metabolism—in relation to SCFA biosynthesis.

Among SCFAs, the metabolic routes involved in acetate, propionate, and butyrate production were identified (Figure 7), as they are commonly found in the human gut [23]. In glycolysis/gluconeogenesis, we found 11 KO IDs across five key enzymes involved in nutrient utilization and ATP supply: pyruvate kinase (EC:2.7.1.40), phosphoenolpyruvate carboxykinase (ATP) (EC:4.1.1.49), pyruvate, and phosphate dikinase (EC:2.7.9.1). These enzymes are important for generating key precursors such as oxaloacetate and pyruvate for acetyl-COA towards acetate and butyrate formation [4,28]. Among SCFAs, acetate is the most abundant SCFA, and it is essential for the growth of other microbes in the human gut. Butyrate is also the main energy source for human colonocytes and enterocytes, and can activate intestinal gluconeogenesis for beneficial effects on glucose and energy homeostasis. In the TCA cycle, we also found NADH supply via only one KO ID and one key enzyme, 2-oxoglutarate synthase (EC:1.2.7.3), across *A. intestini*, *B. dorei*, *B. massiliensis*, *B. vulgatus*, *D. piger*, and *E. siraeum*. This enzyme is interesting because it produces succinyl-COA, which is a precursor for further propionate formation [22,29]. Consistent with other populations from earlier reports, propionate is commonly produced by Bacteroidetes, including *Bacteroides*, for example, *B. vulgatus* through the succinate pathway [30] when treatment with CMH [31].

For pentose and glucuronate interconversions, we observed three KO IDs and three key enzymes involved in NADH supply: l-iditol 2-dehydrogenase (EC: EC:1.1.1.14). This enzyme is widely distributed and has been described in archaea and bacteria across five bacterial species (i.e., *A. hadrus*, *C. saccharolyticum*, *E. coli*, *R. hominis* and *R. intestinalis*). This enzyme is widely distributed in the bacteria. It acts on a number of sugar alcohols, such as D-xylitol, which increases the concentration of propionate, as seen in *E. coli* [32].

For the remaining C5-branched dibasic acid metabolism and propanoate metabolism, we observed two KO IDs and two key enzymes involved in NADH and precursor supply (i.e., 1,2 propanediol) for propionate, 3-isopropylmalate dehydrogenase (EC:1.1.1.85), and methylglyoxal synthase (EC:4.2.3.3), respectively. As previously reported, propanediol-associated pathway carriers almost exclusively belong to the family *Lachnospiraceae*, mainly from the genera *Ruminococcus* and *Blautia* [33,34]. In addition, propionate can be converted to glucose via intestinal gluconeogenesis. Propionate has antibacterial and anti-inflammatory functions that protect the human intestine against pathogens [28].

Beyond integrative metagenomics throughout this study, the predominant species and potential metabolic routes were revealed to be cooperative microbiome networks under different sugar utilizations (e.g., glucose, mannose, or xylose) in relation to SCFA biosynthesis. Taken with tCMH, these results suggest that CMH is a potential prebiotic for human gut microbiome modulation and maintenance.

## 4. Conclusions

Our integrative metagenomics approach under different treatment regimens was enabled using 16S rRNA gene and WMGS sequencing technology. The data were analyzed using bioinformatics and systems biology. Upon treatment with CMH, we positively identified twelve predominant bacterial species, potential metabolic functions and associated routes. Cooperative microbiome networks under sugar utilization (e.g., glucose, mannose, or xylose) and energy supply (i.e., ATP and NADH) engage in central carbon metabolism for biosynthesizing SCFAs, such as propionate, butyrate, and acetate. These findings suggest that CMH may be a potential prebiotic for modulating the gut microbiome. Therefore, this study sheds light on the gut microbiome–metabolism axis implicated in human health.

## Figures and Tables

**Table 1:** Summary of WMGS datasets for each group.

Groups	Raw Reads (Mb)	Clean Reads (Mb)	Effective Rates * (%)
Baseline			
bPB	273.40 ^#^	271.29	99.23
bCMH	276.56 ^#^	273.71	98.97
Treatment			
tPB	301.76	299.58	99.28
tCMH	290.02	287.83	99.24
Total	1141.74	1132.41	99.18

Note: * Means of effective rates are presented. ^#^ Data was taken from Raethong et al. 2021, [11]. In addition, bPB, bCMH, tPB, tCMH represent baseline placebo, baseline CMH, treatment with placebo, treatment with CMH, respectively.

## Data Availability

Raw sequencing data are available in the National Center for Biotechnology Information Sequence Read Archive (NCBI-SRA) repository under the BioProject accession number PRJNA596624. The 16S rRNA gene sequences of the human gut microbiota are available at GenBank: PRJNA709129.

## Supplementary Materials

The following are available online at https://www.mdpi.com/article/10.3390/biology12010021/s1, Supplementary File S1.1: Demographic characteristics of study participants, Supplementary File S1.2: Relative abundance of taxonomic phyla across baselines and treatments. Supplementary File S1.3: Relative abundances of taxonomic families across baselines and treatments. Supplementary File S1.4: A number of KO IDs involved in Glycolysis/Gluconeogenesis, Propanoate metabolism and C5-Branched dibasic acid metabolism between tPB and tCMH groups. Supplementary File S2.1: Summary of WMGS datasets for each group. Supplementary File S2.2: Relative abundance of top 15 taxonomic families across baselines and treatments. Supplementary File S2.3: Relative abundance between tPB and tCMH on eight potential species. Supplementary File S2.4: List of assigned KO IDs with metabolic functions based on KEGG Orthology (KO) database across all groups. Supplementary File S2.5: List of assigned KO IDs with metabolic functions in carbohydrate metabolism based on KEGG Orthology (KO) database between tPB and tCMH groups. Supplementary File S2.6: List of metabolic pathways in carbohydrate metabolism between tPB and tCMH groups. Supplementary File S2.7: A number of KO IDs involved in TCA cycle and pentose and glucuronate interconversions between tPB and tCMH groups. Supplementary File S2.8: A list of bacterial species underlying significant KO IDs between tPB and tCMH groups.

## References

[1] Goodrich J., Waters J., Poole A., Sutter J., Koren O., Blekhman R., Beaumont M., Treuren W., Knight R., Bell J. (2014). Human Genetics Shape the Gut Microbiome. Cell.

[2] Yatsunenko T., Rey F.E., Manary M.J., Trehan I., Dominguez-Bello M.G., Contreras M., Magris M., Hidalgo G., Baldassano R.N., Anokhin A.P. (2012). Human gut microbiome viewed across age and geography. Nature.

[3] Ashaolu T.J., Ashaolu J.O., Adeyeye S.A.O. (2021). Fermentation of prebiotics by human colonic microbiota in vitro and short-chain fatty acids production: A critical review. J. Appl. Microbiol..

[4] Holscher H. (2017). Dietary Fiber and Prebiotics and the Gastrointestinal Microbiota. Gut Microbes.

[5] Gibson G.R., Hutkins R., Sanders M.E., Prescott S.L., Reimer R.A., Salminen S.J., Scott K., Stanton C., Swanson K.S., Cani P.D. (2017). Expert consensus document: The International Scientific Association for Probiotics and Prebiotics (ISAPP) consensus statement on the definition and scope of prebiotics. Nat. Rev. Gastroenterol. Hepatol..

[6] Slavin J. (2013). Fiber and prebiotics: Mechanisms and health benefits. Nutrients.

[7] Intaratrakul K., Nitisinprasert S., Nguyen T.-H., Haltrich D., Keawsompong S. (2022). Manno-oligosaccharides from copra meal: Optimization of its enzymatic production and evaluation its potential as prebiotic. Bioact. Carbohydr. Diet. Fibre.

[8] Prayoonthien P., Rastall R.A., Kolida S., Nitisinprasert S., Keawsompong S. (2019). In vitro fermentation of copra meal hydrolysate by human fecal microbiota. 3 Biotech.

[9] Sathitkowitchai W., Suratannon N., Keawsompong S., Weerapakorn W., Patumcharoenpol P., Nitisinprasert S., Nakphaichit M. (2021). A randomized trial to evaluate the impact of copra meal hydrolysate on gastrointestinal symptoms and gut microbiome. PeerJ.

[10] Nakphaichit M. (2014). Effect of Increasing Dietary Protein from Soybean Meal on Intestinal Microbiota and Their Fatty Acids Production in Broiler Chicken. Adv. Anim. Vet. Sci..

[11] Raethong N., Nakphaichit M., Suratannon N., Sathitkowitchai W., Weerapakorn W., Keawsompong S., Vongsangnak W. (2021). Analysis of human gut microbiome: Taxonomy and metabolic functions in Thai adults. Genes.

[12] Douglas G.M., Maffei V.J., Zaneveld J.R., Yurgel S.N., Brown J.R., Taylor C.M., Huttenhower C., Langille M.G.I. (2020). PICRUSt2 for prediction of metagenome functions. Nat. Biotechnol..

[13] Patumcharoenpol P., Nakphaichit M., Panagiotou G., Senavonge A., Suratannon N., Vongsangnak W. (2021). MetGEMs Toolbox: Metagenome-scale models as integrative toolbox for uncovering metabolic functions and routes of human gut microbiome. PLoS Comput. Biol..

[14] Oksanen J., Blanchet F.G., Friendly M., Kindt R., Legendre P., McGlinn D., Minchin P.R., O’hara R.R., Simpson G.L., Solymos P. (2019). Vegan: Community Ecology Package. https://CRAN.R-project.org/package=vegan.

[15] Wickham H. (2009). ggplot2: Elegant Graphics for Data Analysis.

[16] Truong D.T., Franzosa E.A., Tickle T.L., Scholz M., Weingart G., Pasolli E., Tett A., Huttenhower C., Segata N. (2015). MetaPhlAn2 for enhanced metagenomic taxonomic profiling. Nat. Methods.

[17] Beghini F., McIver L.J., Blanco-Míguez A., Dubois L., Asnicar F., Maharjan S., Mailyan A., Manghi P., Scholz M., Thomas A.M. (2021). Integrating taxonomic, functional, and strain-level profiling of diverse microbial communities with bioBakery 3. eLife.

[18] Kanehisa M., Sato Y. (2020). KEGG Mapper for inferring cellular functions from protein sequences. Protein Sci..

[19] Kanehisa M., Sato Y., Kawashima M. (2022). KEGG mapping tools for uncovering hidden features in biological data. Protein Sci..

[20] Kanehisa M., Sato Y., Kawashima M., Furumichi M., Tanabe M. (2016). KEGG as a reference resource for gene and protein annotation. Nucleic Acids Res..

[21] La-Ongkham O., Nakphaichit M., Nakayama J., Keawsompong S., Nitisinprasert S. (2020). Age-related changes in the gut microbiota and the core gut microbiome of healthy Thai humans. 3 Biotech.

[22] Nogal A., Valdes A.M., Menni C. (2021). The role of short-chain fatty acids in the interplay between gut microbiota and diet in cardio-metabolic health. Gut Microbes.

[23] Portincasa P., Bonfrate L., Vacca M., De Angelis M., Farella I., Lanza E., Khalil M., Wang D.Q.-H., Sperandio M., Di Ciaula A. (2022). Gut Microbiota and Short Chain Fatty Acids: Implications in Glucose Homeostasis. Int. J. Mol. Sci..

[24] Tsukuda N., Yahagi K., Hara T., Watanabe Y., Matsumoto H., Mori H., Higashi K., Tsuji H., Matsumoto S., Kurokawa K. (2021). Key bacterial taxa and metabolic pathways affecting gut short-chain fatty acid profiles in early life. ISME J..

[25] Louis P., Flint H.J. (2017). Formation of propionate and butyrate by the human colonic microbiota. Environ. Microbiol..

[26] Palmas V., Pisanu S., Madau V., Casula E., Deledda A., Cusano R., Uva P., Vascellari S., Loviselli A., Manzin A. (2021). Gut microbiota markers associated with obesity and overweight in Italian adults. Sci. Rep..

[27] Mirzaei R., Bouzari B., Hosseini-Fard S.R., Mazaheri M., Ahmadyousefi Y., Abdi M., Jalalifar S., Karimitabar Z., Teimoori A., Keyvani H. (2021). Role of microbiota-derived short-chain fatty acids in nervous system disorders. Biomed. Pharmacother..

[28] Markowiak-Kopeć P., Śliżewska K. (2020). The Effect of Probiotics on the Production of Short-Chain Fatty Acids by Human Intestinal Microbiome. Nutrients.

[29] Macy J.M., Ljungdahl L.G., Gottschalk G. (1978). Pathway of succinate and propionate formation in Bacteroides fragilis. J. Bacteriol..

[30] Flint H.J., Scott K.P., Louis P., Duncan S.H. (2012). The role of the gut microbiota in nutrition and health. Nat. Rev. Gastroenterol. Hepatol..

[31] Prayoonthien P., Nitisinprasert S., Keawsompong S. (2018). In vitro fermentation of copra meal hydrolysate by chicken microbiota. 3 Biotech.

[32] Xiang S., Ye K., Li M., Ying J., Wang H., Han J., Shi L., Xiao J., Shen Y., Feng X. (2021). Xylitol enhances synthesis of propionate in the colon via cross-feeding of gut microbiota. Microbiome.

[33] Kircher B., Woltemate S., Gutzki F., Schlüter D., Geffers R., Bähre H., Vital M. (2022). Predicting butyrate- and propionate-forming bacteria of gut microbiota from sequencing data. bioRxiv.

[34] Van der Hee B., Wells J.M. (2021). Microbial Regulation of Host Physiology by Short-chain Fatty Acids. Trends Microbiol..

